# Novel Mutations Evading Avian Immunity around the Receptor Binding Site of the Clade 2.3.2.1c Hemagglutinin Gene Reduce Viral Thermostability and Mammalian Pathogenicity

**DOI:** 10.3390/v11100923

**Published:** 2019-10-09

**Authors:** Se-Hee An, Chung-Young Lee, Seung-Min Hong, Chang-Seon Song, Jae-Hong Kim, Hyuk-Joon Kwon

**Affiliations:** 1 Laboratory of Avian Diseases, College of Veterinary Medicine, Seoul National University, Seoul 08826, Korea; eepdl1201@snu.ac.kr (S.-H.A.); topkin@snu.ac.kr (S.-M.H.); kimhong@snu.ac.kr (J.-H.K.); 2 Department of Microbiology and Immunology, Emory University School of Medicine, 1510 Clifton Road, Atlanta, GA 30322, USA; chung-young.lee@emory.edu; 3 Laboratory of Avian Diseases, College of Veterinary Medicine, Konkuk University, Seoul 05029, Korea; songcs@konkuk.ac.kr; 4 Research Institute for Veterinary Science, College of Veterinary Medicine, Seoul National University, Seoul 08826, Korea; 5 Laboratory of Poultry Medicine, Department of Farm Animal Medicine, College of Veterinary Medicine, Seoul National University, Seoul 08826, Korea; 6 Farm Animal Clinical Training and Research Center (FACTRC), GBST, Seoul National University, Kangwon-do 88026, Korea

**Keywords:** clade 2.3.2.1c H5N1 virus, immunity evasion, HA trimer stability, thermostability, mammalian pathogenicity

## Abstract

Since 2007, highly pathogenic clade 2.3.2 H5N1 avian influenza A (A(H5N1)) viruses have evolved to clade 2.3.2.1a, b, and c; currently only 2.3.2.1c A(H5N1) viruses circulate in wild birds and poultry. During antigenic evolution, clade 2.3.2.1a and c A(H5N1) viruses acquired both S144N and V223I mutations around the receptor binding site of hemagglutinin (HA), with S144N generating an *N*-glycosylation sequon. We introduced single or combined reverse mutations, N144S and/or I223V, into the HA gene of the clade 2.3.2.1c A(H5N1) virus and generated PR8-derived, 2 + 6 recombinant A(H5N1) viruses. When we compared replication efficiency in embryonated chicken eggs, mammalian cells, and mice, the recombinant virus containing both N144S and I223V mutations showed increased replication efficiency in avian and mammalian hosts and pathogenicity in mice. The N144S mutation significantly decreased avian receptor affinity and egg white inhibition, but not all mutations increased mammalian receptor affinity. Interestingly, the combined reverse mutations dramatically increased the thermostability of HA. Therefore, the adaptive mutations possibly acquired to evade avian immunity may decrease viral thermostability as well as mammalian pathogenicity.

## 1. Introduction

Highly pathogenic H5N1 avian influenza A (HP A(H5N1)) viruses are fatal to poultry and cause high human fatality after dead-end transmission from infected poultry [1,2]. HP A(H5N1) viruses spread from Asia to Africa and Europe by migratory birds, and antigenic evolution has continued under immune pressure by vaccination and natural infection in Asia [1,3,4]. The ancestral HP A(H5N1) virus A/goose/Guangdong/1/96 (clade 0) has evolved into multiple clades from clade 1 to 9 [5]. Some clade 2.3.2 viruses evolved into clade 2.3.2.1 and further diversified into 2.3.2.1a, b, and c in 2009 [6,7]. Clade 2.3.2.1c viruses have spread from Far East and South East Asian countries to Dubai, Bulgaria, Romania, and Nigeria and have become enzootic in Asian countries [8,9,10,11].

Hemagglutinin (HA) is a surface glycoprotein exposed on the outside of virus particles and it forms a noncovalent homotrimer composed of a distal globular head and proximal stalk [12]. The receptor binding site (RBS) on the globular head of HA is a shallow pocket-like structure consisting of three secondary structure elements (130-loop, 190-helix, and 220-loop) and a base (Y98, W153, H183, and Y195 in H3 numbering) [12]. HA binds to cell surface receptors to infect the host cell, and avian and human influenza A viruses (IAVs) preferentially bind to sialic acid α2,3-linked (α2,3 SA) and α2,6-linked (α2,6 SA) to galactose in avian and mammalian receptors, respectively. However, mutations in the RBS of avian IAVs can change receptor affinity from affinity only to α2,3 SA to both α2,3 SA and α2,6 SA or only α2,6 SA to overcome host barriers, resulting in interspecies transmission and adaptation [13,14,15]. Several mutations that increase pathogenicity and affinity to mammalian receptors have been reported in the 220-loop of the H5 subtype HA (Q226L, G228S, etc.) [16].

The globular head of HA is a major target of humoral immunity and is a hotspot of cumulative missense mutations to escape host immune responses [17,18,19,20]. Epitope mapping and escape mutant studies with mouse monoclonal antibodies have revealed antigenic variations of H5N1 IAVs [18,19]. The evasion mutations identified in H5 were distributed in epitope sites A (140–145 residues, H3 numbering) and B (155–166, H3 numbering) of H3 and Sa of H1 (129–133, H3 numbering) [18,19,21,22]. Acquisition of an N-glycosylation sequon (NGS) in the epitope not only shields the epitopes from antibody binding but also affects the binding affinity of HA to mammalian receptors [16,23].

The HA trimer is stabilized by polar and nonpolar interactions between the three stems and intermolecular salt bridges between the globular heads at low pH [24,25,26]. The H103Y and T318I mutations increase low pH stability and thermostability, as well as droplet transmission of H5N1 viruses between ferrets [15,16,27,28]. Therefore, multiple mutations of HA acquired in a stepwise manner that play roles in receptor affinity, immunity evasion, and structural/functional stability may cooperatively affect the mammalian pathogenicity of avian IAVs.

Therefore, in this study, we compared the HA amino acid sequences of clade 2.3.2 and clade 2.3.2.1a, b, and c viruses and found two cumulative mutations (S144N and V223I) around the RBS. Most clade 2.3.2.1a and c viruses acquired both mutations. Previously, the PR8-based recombinant virus with HA and Neuraminidase (NA) of a clade 2.3.2.1c HP A(H5N1) virus isolated in Korea, A/mandarin duck/Korea/K10-483/2010 (K10-483), did not replicate well in embryonated chicken eggs (ECEs) and had low pathogenicity in mice [29,30]. To understand the effects of the mutations on replication efficiency in ECEs and mammalian cells and on the pathogenicity in mice, we generated PR8-derived mutant viruses and compared their biological characteristics. In addition, we compared the effects of the mutations on egg white resistance and thermostability of HA.

## 2. Materials and Methods

### 2.1. Viruses, Plasmids, Cells, and Eggs

The attenuated HA (mutation from multi-basic RERRRKR to mono-basic ASGR) and NA genome segments of a clade 2.3.2.1c HP A(H5N1) virus, A/mandarin duck/Korea/K10-483/2010 (K10-483), were previously cloned into a bidirectional reverse genetics vector, pHW2000, and six other internal genomes of A/Puerto Rico/8/34 (H1N1) (PR8) cloned into pHW2000 were used [29,31,32]. The amino acid sequences of HA and NA of K10-483 did not have known mutations affecting the biological traits tested in this study. The 293T, MDCK (Madin-Darby Canine Kidney), and A549 cells were purchased from Korean Collection for Type Cultures (KCTC, Daejeon, Korea). The 293T and MDCK cells were maintained in DMEM supplemented with 10% FBS (Life Technologies Co., Carlsbad, CA, USA), and A549 cells were maintained in DMEM/F12 supplemented with 10% FBS. Virus was propagated with ten-day-old SPF (Specific Pathogen Free) embryonated chicken eggs (ECEs, Charles River Lab., Willimantic, CT, USA).

### 2.2. Data Mining and Analysis of HA Genes and In Silico Analysis of HA Trimer Structure and N-Glycan Profiles

The HA gene sequences of clade 2.3.2, clade 2.3.2.1, and clades 2.3.2.1a, b, and c HP A(H5N1) viruses were collected from the Influenza Virus Database (https://www.ncbi.nlm.nih.gov/genomes/FLU/Database/nph-select.cgi?go=database) and Global Initiative on Sharing All Influenza Data (GISAID, https://www.gisaid.org/) (*n* = 647) on April 14, 2018. The collected nucleotide sequences were translated and compared with the BioEdit program (ver. 7.2.5). Additionally, HA sequences of all A(H5) viruses were collected (*n* = 4189), and amino acid sequences and frequencies of 144NGS and 158NGS and residue 223 (V or I) were analyzed. In addition, HA genes (*n* = 513) of HP A(H5N1) strains from laboratory-confirmed human cases were analyzed as above. Residues 144N and 223V/I were localized and analyzed for intermolecular interactions with other residues in the 3D structure of the H5 trimer (modified 4juk.pdb and 6e7g.pdb) using PyMOL (Molecular Graphis System version 2.3.1, Delano Scientific LLC, South San Francisco, CA, USA). The *N*-glycosylation prediction at position 144 was performed by the NetNGlyc program (DTU Bioinformatics, Lyngby, Denmark).

### 2.3. Site-Directed Mutagenesis and Generation of Viruses by Reverse Genetics

To generate mutated H5N1 recombinant viruses, the cloned HA genome of K10-483 was mutated with a Muta-direct Site Directed Mutagenesis Kit (iNtRON, Gyeonggi, Korea) and specific primer sets (Table 1). A Hoffmann’s reverse genetics system with a few modifications was used for recombinant virus generation [32]. Briefly, 300 ng of each of the eight plasmids were transfected together into confluent 293T cells in 6-well plates (10^6^ cells/well) with Lipofectamine 2000 and PLUS reagents (Life Technologies Co.). After overnight incubation, 1 mL of Opti-MEM (Life Technologies Co.) and 1 µg/mL of TPCK-treated trypsin (Sigma-Aldrich, St. Louis, MO, USA) were added to transfected cells. The supernatant was harvested after another overnight incubation, and 200 μL of the supernatant was inoculated into ten-day-old ECEs. The presence of the recombinant viruses in allantoic fluids was checked by HA assay according to the World Health Organization (WHO) Manual on Animal Influenza Diagnosis and Surveillance, and the genome segments were confirmed by RT-PCR and sequencing as previously described [33].

### 2.4. Comparative Replication Efficiency in ECEs and Growth Kinetics in MDCK and A549 Cells

The generated recombinant viruses (E1) were passaged in ten-day-old SPF ECEs, and the titers of the recombinant viruses (E2) were measured to obtain the 50% chicken embryo infection dose (EID_50_). Each virus was diluted 10-fold and inoculated into ECEs. Virus replication was confirmed by a plate hemagglutination test with 1.0% chicken Red Blood Cells (RBCs), and the EID_50_ was calculated by the Spearman–Karber method [34]. To compare the replication efficiency of the recombinant viruses, the same number of viruses were used to infect ECEs and mammalian cells. One hundred EID_50_ of recombinant virus (E2) were inoculated into ten-day-old SPF ECEs, and after 3 days, the EID_50_ of harvested allantoic fluid was measured as above. In MDCK and A549 cells, 10^5^ EID_50_ of each virus were used to infect confluent MDCK and A549 cells in 12-well plates. The supernatant of the infected cells was harvested at 0, 12, 24, 48, and 72 h post inoculation (hpi), and 10-fold diluted supernatant was used to inoculate confluent MDCK cells in a a 96-well plate to measure the 50% tissue culture infectious dose (TCID_50_) at each time point. Virus replication was confirmed by a hemagglutination assay, and TCID_50_ was calculated using the Spearman–Karber method as above.

### 2.5. Mouse Pathogenicity Test

The mouse pathogenicity test was approved on January 10, 2019 by the Institutional Animal Care and Use Committee (IACUC) of Seoul National University (IACUC-SNU-171214-1-1). The approved experiment was performed in a biosafety level 2 facility at the Animal Center for Pharmaceutical Research of Seoul National University (Seoul, Korea) according to the national guidelines for the care and use of laboratory animals. Six-week-old female BALB/c mice (*n* = 8) (KOATEC, Pyeongtaek, Korea) were anesthetized by Zoletil 50 (15 mg/kg, IP) (Virbac, Carros, France), and 10^6^ EID_50_/50 μL of each recombinant virus was inoculated intranasally. Five mice from each group were weighed for 2 weeks, and three mice were euthanized to obtain lung samples at 3 days post inoculation (dpi). During the experiment, mice with 20% or more body weight loss were euthanized. The sampled lungs were ground with TissueLyzer 2 and 5 mm stainless steel beads (Qiagen, Valencia, CA, USA) and suspended in PBS. The virus titer (EID_50_) was measured as previously described [35].

### 2.6. Solid-Phase Receptor Binding Assays

To evaluate the receptor binding affinity of recombinant viruses, a solid-phase assay was used as previously described with some modifications [36,37]. In short, 96-well enzyme-linked immunosorbent assay plates (SPL, Gyeonggi, Korea) coated with 10 µg/mL fetuin (Sigma-Aldrich) were bound with the recombinant viruses overnight. After washing the virus-bound plates three times with PBS + 0.05% Tween 20 (PBST), the plates were blocked with 0.1% desialylated BSA + 10 µM oseltamivir (Sigma-Aldrich) for 1 h at 4 °C. The blocked plates were washed three more times with PBST, and the biotinylated sialylglycopolymers (Neu5Acα2-3Galb1-4GlcNAcb-PAA-biotin, 3′SLN-PAA, and Neu5Acα2-6GalNAca-PAA-biotin, 6′SLN-PAA) (Glycotech Corporation, Gaithersburg, MD, USA) were serially diluted and added to the plates for 1 h at 4 °C. Then, the plates were washed three times with PBST and incubated with horseradish peroxidase (HRP)-conjugated streptavidin (Thermo Fisher Scientific, Waltham, MA, USA) for 1 h at 4 °C. Finally, HRP was developed with 3,3’5,5’-Tetramethylbenzidine (TMB) substrate (SurModics, Eden Prairie, MN, USA), and the chromogenic reaction was stopped by adding 0.1 M sulfuric acid. The absorbance at 450 nm was measured by a microplate reader (TECAN, Männedorf, Switzerland).

### 2.7. HA and Hemagglutination Inhibition (HI) Tests

The HA test and HI test with chicken RBCs and guinea-pig RBCs were performed according to the WHO manual for the laboratory diagnosis and virological surveillance of influenza. The recombinant virus was serially diluted 2-fold in 96-well plates, and chicken RBCs (1%) or guinea-pig RBCs (1%) were added. After 40 min of incubation at 4 °C, the hemagglutination unit (HAU) of each virus was recorded. Chicken RBCs have similar amounts of sialic acids bound to galactose by α2,3 linkage (SAα2,3Gal) and sialic acid linked to galactose by α2,6 linkage (SAα2,6Gal), and guinea pig RBCs have more SAα2,6Gal than SAα2,3Gal [38]. The HI test of recombinant viruses was conducted with chicken egg white to compare the resistance of the recombinant viruses against egg white. Chicken egg white was serially diluted as in the HA test, and 4 HAU of each virus were added to each well and incubated for 30 min at 4 °C. Then, chicken RBCs or guinea pig RBCs were added, and the HI titer was recorded after 40 min of incubation at 4 °C. All experiments were repeated three times independently.

### 2.8. Heat Stability Test

Recombinant viruses were diluted to the same HA titer (2^4^) and aliquoted for heat treatment. Each aliquot was incubated at 60 °C for 0, 5, 15, and 30 min, and the HA titer was measured.

### 2.9. SDS-PAGE and Western Blotting

To confirm the 144N glycosylation, 4 µL of each recombinant virus (CE3) treated or untreated with PNGase F enzyme (New England Biolabs, Ipswich, MA, USA) was mixed with Protein 5X Sample Buffer (ELPIS BIOTECH, Daejeon, Korea) to denature it for 5 min at 95 °C, and SDS-PAGE was performed using NuPAGE 4–12% Bis-Tris Protein Gels (Life Technologies Co.). The proteins were transferred to a nitrocellulose membrane (Life Technologies Co.), and the membrane was incubated with anti-H5N1 virus (A/Vietnam/1194/2004), HA rabbit IgG (Sino Biological Inc., Beijing, China), followed by incubation with horseradish peroxidase conjugated-goat anti-rabbit IgG (Bethyl Laboratories Inc., Montgomery, AL, USA). Then, HA proteins were visualized with BioFX TMB One Component HRP Membrane Substrate (SurModics IVD, INC., Eden Prairie, MN, USA) and sulfuric acid stop solution (Sigma-Aldrich).

### 2.10. Statistical Analysis

All data were analyzed with IBM SPSS Statistics version 23 (IBM., Armonk, NY, USA). The statistical significance of viral titers in ECEs, growth kinetics in cells, and receptor binding affinity were evaluated by one-way analysis of variance (*p* < 0.05). Survival rates were compared by Kaplan–Meier survival analysis, and the differences in frequency were assessed by chi-square test (*p* < 0.05).

## 3. Results

### 3.1. Comparison of Amino Acid Sequences of Clade 2.3.2, Clade 2.3.2.1, and Clade 2.3.2.1a, b, and c Proteins

All the amino acid sequences of HA proteins of clade 2.3.2 and clade 2.3.2.1 strains in the databases were compared, and we found variations at 144NGS and 158NGS and at amino acid residue 223 around the RBS (Table 2). We summarized the genetic profiles of representative early strains of each clade in Table 2. The early strains of clade 2.3.2 and clade 2.3.2.1b contained neither 144NGS nor 158NGS, or only 144NGS with 223V in common, but clade 2.3.2.1a (none or only 144NGS) and clade 2.3.2.1c (only 144NGS) did have in common the V223I mutation. The frequencies of 144NGS and 158NGS were similar in strains to clade 2.3.2, but most of the 2.3.2.1a, b, and c viruses had 144NGS rather than 158NGS [39].

### 3.2. Analysis of 144NGS, 158NGS, and Residue 223 Profiles among H5 Sequences in the Database

According to the mutation profiles (MPs) of 144NGS, 158NGS, and residue 223, the H5 sequences deposited in the Influenza Virus Database (*n* = 4189) were classified into 7 MPs (Table 3). MP1-1 characterized by the presence of 223V without either 144NGS or 158NGS was the most frequent (56.7%, wild-type), and MP2-1, containing 158NGS and 223V, was the second most frequent (26.8%). MP3-2, containing 144NGS and 223I, similar to clades 2.3.2.1a and c, was 11.4%. MP1-1 was significantly more frequent than other MPs (*p* < 0.05) (Table 3). We classified the 144–146 and 158–160 amino acid sequences according to the required number of point mutations to become putative NGS (precursor NGS) (Table S1). The frequency of 144NGS + 1 was 16.5% and was less frequent than 158NGS+1 (44.9%). The frequency of 144NGS + 2 was 61.1% and was more frequent than 158NGS + 2 (24.5%). To confirm the preferred selection of 158NGS to 144NGS, we counted the total number (188) of HA genes with both amino acid sequences converting to 144NGS (144NGS + 1) or 158NGS (158NGS + 1) by a single point mutation. In addition, we counted the numbers of genes harboring 144NGS or 158NGS with 158NGS + 1 (117) or 144NGS + 1 (406), respectively. The frequency of 144NGS/158NGS+1 was 16.5% (117/711, the total number is the sum of 188, 117, and 406), and 2.4% (17/711) and 14.1% (100/711) possessed V and I at residue 223, respectively. The frequency of 144NGS+1/158NGS was 57.1% (406/711), and 54.6% (388/711) and 2.5% (18/711) possessed V and I at residue 223, respectively (Table 2). Therefore, 158NGS was significantly more frequent than 144NGS, and 223V was more frequent than 223I in the 144NGS + 1/158NGS group (*p* < 0.05). However, 223I was more frequent than 223V in the 144NGS/158NGS+1 group (*p* < 0.05).

### 3.3. Generation of PR8-Derived Clade 2.3.2.1 H5N1 Recombinant Viruses and Comparison of Viral Replication Efficiency in ECEs

PR8-derived H5N1 recombinant viruses containing single (N144S or I223V) and combined (N144S and I223V) mutations were generated (Table 4). The replication efficiency of the recombinant viruses was compared in terms of 50% chicken embryo infection dose (EID_50_). The virus titer of rH5N1-N144S-I223V (10 ^9.05 ± 0.18^ EID_50_/mL) was significantly higher than those of rH5N1-N144S (10^8.20 ± 0.17^ EID_50_/mL) and rH5N1-V223I (10 ^7.48 ± 0.23^ EID_50_/mL) (*p* < 0.05) but insignificantly higher than that of rH5N1 (Table 4).

### 3.4. Comparison of Replication Efficiency in Mammalian Cells and Mouse Pathogenicity of the H5N1 Recombinant Viruses

**Figure 1:**
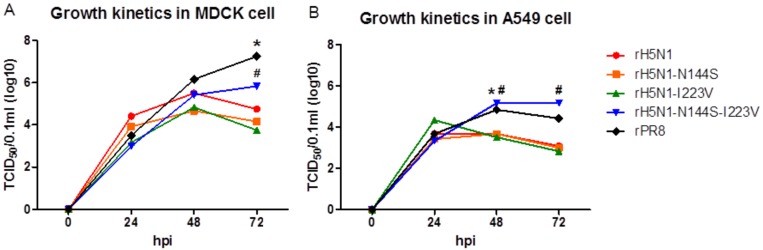
Growth kinetics of recombinant H5N1 viruses in MDCK and A549 cells. Each recombinant virus was diluted to 10^5^ 50% chicken embryo infection dose (EID_50_)/0.1 mL, and 0.5 mL of diluents were inoculated into confluent (**a**) MDCK and (**b**) A549 cells in 6-well plates for 1 h. After 1 h, the inoculated virus was removed, and 1 mL of fresh medium was added. During 72 h of incubation, the supernatant was harvested at 0, 24, 48, and 72 hpi, and 50% tissue culture infectious dose (TCID_50_)/0.1 mL of each time point was measured in MDCK cells. The TCID_50_/0.1 mL values are the average of three independent experiments. #, *, significant differences of rH5N1-N144S-I223V (#) and rPR8 (*) in comparison with other viruses (*p* < 0.05).

The replication efficiency of H5N1 recombinant viruses was compared in MDCK and A549 cells. The viral titers in MDCK cells were similar among the recombinant viruses at 24 and 48 hpi, but rH5N1-N144S-I223V showed a significantly higher titer than other H5N1 recombinant viruses at 72 hpi (Figure 1A). In A549 cells, rH5N1-N144S-I223V showed significantly higher viral titers at 48 and 72 hpi (Figure 1B).

**Figure 2:**
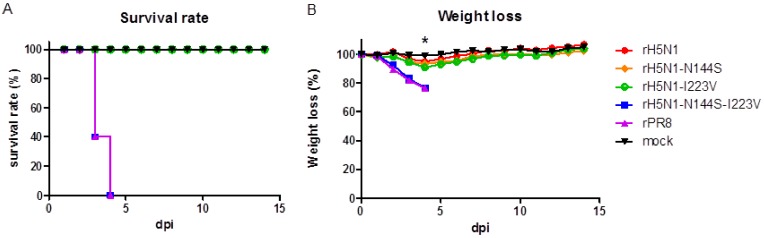
Mouse pathogenicity of recombinant H5N1 viruses. (**a**) Mortality and (**b**) weight loss of mouse experimental groups infected with recombinant H5N1 viruses. Five six-week-old female BALB/c mice per group were inoculated with 10^6^ EID_50_ of virus or an equivalent volume PBS (mock) intranasally. Weight loss was monitored for 2 weeks, and mice with more than 20% weight loss were euthanized. The weight loss was calculated based on the body weight measured at 0 dpi, and the data are the average of each group; * significant difference of the rH5N1-I223V and rH5N1-N144S groups compared to the mock groups (*p* < 0.05).

All the H5N1 recombinant viruses replicated in the lungs of infected BALB/c mice at 3 dpi, but rH5N1-N144S-I223V showed a significantly higher virus titer than that of other H5N1 recombinant viruses (Table 5). In addition, rH5N1-N144S-I223V infection resulted in apparent body weight loss in all mice, and the mice died (100% mortality) within 4 dpi (Figure 2). However, rH5N1-N144S, rH5N1-I223V, and rH5N1 did not cause significant body weight loss during the observation period.

### 3.5. Comparison of Binding Affinity of the H5N1 Recombinant Viruses to Avian (3′-SLN) and Mammalian (6′-SLN) Receptors and Egg White

**Figure 3:**
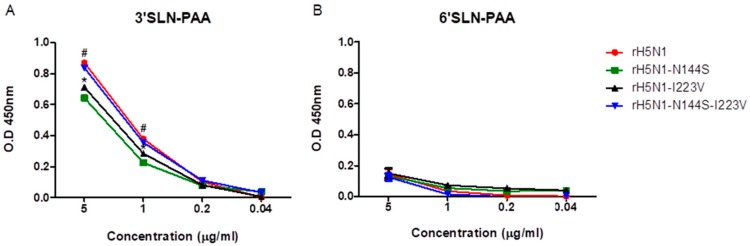
Receptor binding affinity of recombinant H5N1 viruses. The two types of serially diluted biotinylated sialylglycopolymers (Neu5Acα2-3Galb1-4GlcNAcb–PAA-biotin (3′SLN–PAA) and Neu5Acα2-6GalNAca–PAA-biotin (6′SLN–PAA)) were incubated with the same concentration (10^5^ EID_50_) of recombinant viruses. After development with horseradish peroxidase (HRP)-conjugated streptavidin and 3,3’5,5’-Tetramethylbenzidine (TMB) substrate, the reaction was stopped by adding stop solution, and the absorbance at 450 nm was measured. (**a**) Receptor binding affinity of recombinant viruses to 3′SLN–PAA and (**b**) Receptor binding affinity of recombinant viruses to 6′SLN–PAA. The absorbance data are the average of three independent experiments, # significant difference of rH5N1 and rH5N1-N144S-I223V compared to the other viruses, * significant difference compare to rH5N1–N144S (*p* < 0.05).

We compared the receptor binding affinity of recombinant viruses to 3′-SLN and 6′-SLN (Figure 3). All the recombinant viruses bound to 3′-SLN more strongly than to 6′-SLN. rH5N1 and rH5N1-N144S-I223V showed significantly higher binding affinities than other viruses, and rH5N1-N144S had the weakest binding affinity to the avian receptor (Figure 3). When chicken RBCs were used to measure HI titers in egg white for the H5N1 recombinant viruses, only rH5N1-N144S showed slightly lower HI titers than other viruses (64 vs. 128, Table 6). However, the difference was much higher when we used guinea pig RBCs (<8 vs. 64). Therefore, rH5N1-N144S was significantly less inhibited by egg white, and 223I may play a role in the resistance to egg white.

### 3.6. Comparison of Thermostability of the H5N1 Recombinant Viruses

**Figure 4:**
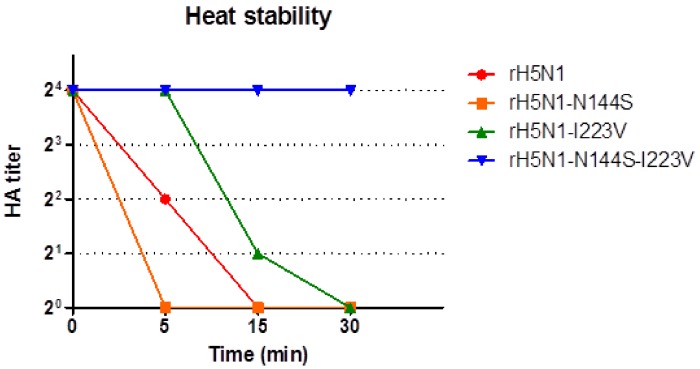
Heat stability of recombinant H5N1 viruses. Each of the recombinant viruses was diluted to a 2^4^ HA titer, and aliquots were incubated at 60 °C for 0, 5, 15, and 30 min. After heat treatment, the HA titer of each aliquot was measured by HA assay with 1% chicken RBCs.

The HA titers of the H5N1 recombinant viruses decreased to zero within 5 (rH5N1-N144S), 15 (rH5N1), and 30 min (rH5N1-I223V) after heat treatment, but that of rH5N1-N144S-I223V was maintained even after heat treatment for 30 min (Figure 4). Therefore, the V223I mutation may decrease HA thermostability.

### 3.7. Intra- and Intermolecular Interactions of Residue 223 in the HA Trimer and Confirmation of the 144N-Glycosylation

**Figure 5:**
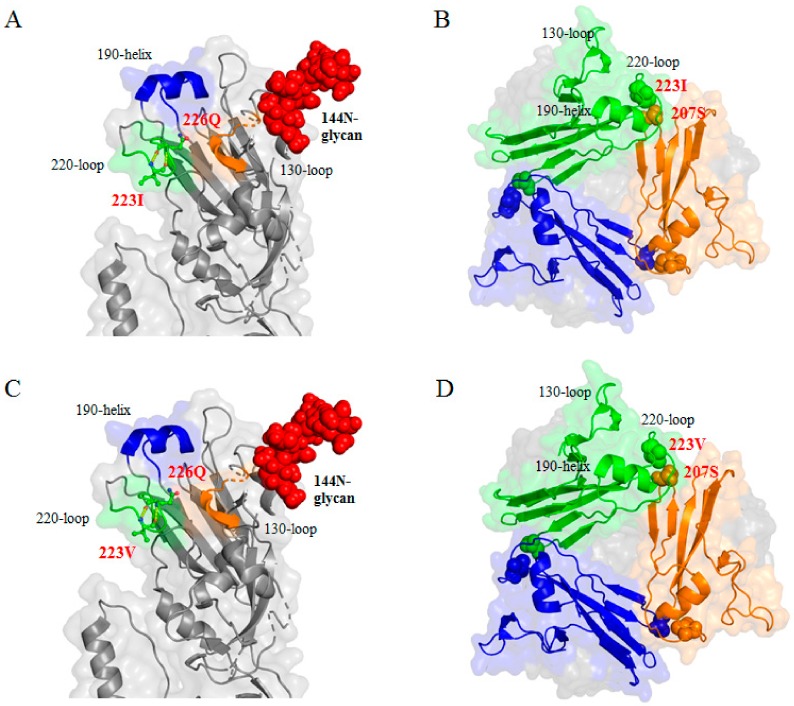
Location and intermolecular interaction of 144N and 223V/I residues in the 3D structure of the HA trimer. HA and HA trimer structure were modified from 4juk.pdb and 6e7g.pdb using PyMOL. (**a**) 223I and (**c**) 223V were located in the 220-loop of the receptor binding site (RBS) of the globular head. Position 223 was close to position 226Q, and both 223I and 223V interacted with 226Q by hydrogen bond (dotted line). (**a**), (**c**) 144N followed by 145S and 146S were located near the RBS, and 144N glycosylation was formed by N-X-S/T. (**b**) 223I and (**d**) 223V were close to the 207S of another HA monomer, and 223I had more side chains extruding and was much closer to 207S than 223V.

**Figure 6:**
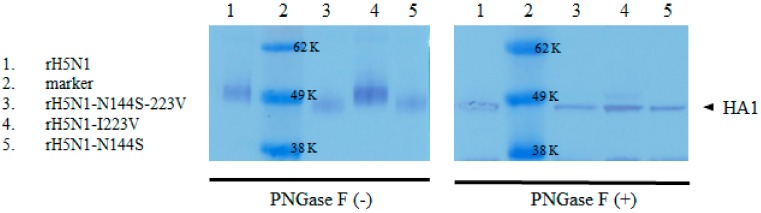
Verification of 144N-glycosylation by Western blotting. Recombinant viruses untreated and treated with PNGase F enzyme were denatured and separated by SDS-PAGE. Transferred membranes were incubated with rabbit anti-influenza A H5N1 (A/Vietnam/1194/2004) HA IgG, followed by goat anti-rabbit IgG HRP-conjugated secondary antibody. Then, HRP was developed by TMB substrate. HA proteins of rH5N1 and rH5N1-I223V had 144N-glycan, and they had higher molecular weight than rH5N1-N144S and rH5N1-N144S-I223V in the absence of PNGase F enzyme treatment (−). However, the difference disappeared after treatment of PNGase F enzyme (+).

Residue 223 is located in the 220-loop, and both 223I and 223V interact intramolecularly with 226Q via hydrogen bonding. Interestingly, residue 223 is located close to 207S of another neighboring HA monomer. Considering the larger side chain of I than V, the V223I mutation may affect the integrity of the HA trimer (Figure 5). So far, 144NGS is considered to be a real N-glycosylation site, and our Western blotting data also agree with previous reports (Figure 6) [44].

## 4. Discussion

Clade 2.3.2 viruses were isolated from ducks and wild birds in mainland China and Hong Kong in 2004, but they might have been already present in mainland China in 2003 due to virus isolation from Muscovy ducks smuggled from China to Taiwan [6,39,45]. New clade 2.3.2.1 viruses derived from clade 2.3.2 viruses appeared in 2007, and clades 2.3.2.1a, b, and c viruses appeared in 2009 [7,45]. In Viet Nam, clades 2.3.2.1a, b, and c viruses used to cocirculate, but clade 2.3.2.1b viruses have disappeared [10]. Clade 2.3.2.1 viruses acquired 144NGS, and this mutation was conserved in most of the clade 2.3.2.1a, b, and c viruses. Considering the significantly higher frequency of 158NGS than 144NGS among A(H5) viruses, given the same possibility of acquiring either 144NGS or 158NGS (Table S1), the acquisition of 144NGS may reflect the presence of certain selection pressure or other circumstances.

HA glycosylation to evade humoral immunity may be the most effective but final choice because it reduces viral fitness [46]. The variability of NGS precursors and their higher frequencies than NGS at 144–146 (86.5% vs. 13.5%) and 158–160 (71.9% vs. 28.1%) may be in line with the above notion (Table S1). The 144N-glycan reduced the replication efficiency in ECEs and the a2,3 SA affinity of rH5N1-I223V in comparison with rH5N1-N144S-I223V. Therefore, 144N-glycan in H5 reduced viral fitness, similar to the findings in a previous report in H1 [46]. However, circumstantial evidence explaining why clade 2.3.2.1 viruses chose 144NGS instead of the more prevalent and preferable 158NGS needs to be discussed further.

Humoral immunity induced by vaccination may facilitate the appearance of mutants evading vaccine immunity [47]. The vaccine program was implemented with A/chicken/Mexico/232-CPA/1994 (H5N2) in Hong Kong (HK) from 2002 to 2003 and with A/turkey/England/N28/1973 (H5N2) from 2004 to 2006 in mainland China. Monovalent (Re-1 (clade 1) from 2004 to 2008, Re-4 (clade 7.2) from 2006 to 2012, Re-5 (clade 2.3.4) from 2008 to 2012, Re-6 (clade 2.3.2) in 2012) and bivalent (Re-1/Re-4 from 2007 to 2008, Re-4/Re-5 from 2008 to 2012 and Re-4/Re-6 in 2012) inactivated PR8-derived recombinant vaccines have been used [48,49]. Among them, Re-1, Re-4, and Re-5 were the major vaccines in mainland China during the evolution period (2005–2009) of clade 2.3.2 to clades 2.3.2.1a, b, and c, and Re-4 and Re-5 contained only 158NGS, and other viruses, except Re-6 (only 144NGS), contained neither 144NGS nor 158NGS [50]. Therefore, vaccine-induced antibodies might have targeted the shielded epitope site B (group 2, 155–166) rather than epitope site A (group 1, 140–145), and mutant viruses acquiring 144NGS to shield epitope site B might have been selected [44]. Re-6 with 144NGS might have been effective against clade 2.3.2.1a, b, and c viruses not only due to antigenic similarity but also due to the restricted acquisition of additional 158NGS. The very low frequency of A(H5) viruses possessing both 144NGS and 158NGS (0.1%) may reflect the inferior competitiveness of such HAs in nature (Table 3). In Viet Nam, where clade 2.3.4 and clade 2.3.2.1a, b, and c viruses had cocirculated, Re-1, from 2005 to 2010, and Re-5, (clade 2.3.4) since 2011, had been used for vaccinations [51]. Clade 2.3.4 viruses declined after vaccination, but clade 2.3.2.1c viruses became enzootic, possibly due to antigenic mismatch and the shielding effect of 144N-glycan [10,52,53].

The V223I mutation is unlikely to stand alone without 144NGS due to its very low frequency in nature, and stepwise acquisitions of 144NGS and V223I mutations during clade 2.3.2.1a, b, and c diversification from clade 2.3.2.1 are noteworthy (Table 3). Similarly, cooperating mutations with N-glycans, K147 with 144NGS, and N227S with 158NGS have been reported [23,54]. The biological effects and roles of the V223I mutation are unclear, but a single V223I mutation decreased the virus replication efficiency of rH5N1-N144S in ECEs. Additionally, rH5N1-N144S showed resistance to egg white due to a relatively steep decrease in HI titer and significantly lower a2,3 SA affinity than those of the other viruses in this study (Table 6, Figure 3). Ovomucin in egg white is an effector molecule of innate immunity present on the surface of the mucous membrane, and a different mutation reducing the inhibition has been reported [36,55]. Therefore, characterization of egg white resistance may be useful to understand the evolutionary status of IAVs. Mutations such as S223N (S224N according to our H3 numbering), N224K, G225D, Q226L, G228S, and S227N directly or indirectly increase a2,6 SA affinity, and their side chains are located in or near the RBS. However, the side chain of residue 223 is located at the interface of the globular heads of the HA trimer (Figure 5). The amino acid residues located between the interfaces of the HA trimer affect structure and pH stability, and the electrostatic intermolecular interaction between T212 and N216 and the increased rigidity of the S221P mutation stabilized the HA trimer to increase pH stability [25,26]. The lower thermostability of rH5N1-N144S than rH5N1 and rH5N1-I223V may imply a negative effect of the intermolecular interaction between 223I and 207S on HA thermostability (Figure 5). V/I223 did not interact with S207 via hydrogen bonding, and the bulkier side chain of I may cause steric hindrance at the interface. Although we did not test pH stability, a pH stability-related mutation (H103Y) showed increased thermostability by stabilizing the HA trimer in previous reports [56,57]. Therefore, the V223I mutation acquired in addition to S144N during adaptation in vaccinated poultry might have improved viral fitness in terms of a2,3 SA receptor affinity at the cost of decreased HA trimer stability.

The mammalian pathogenicity of HP A(H5N1) viruses is a multigenic trait, and the human pathogenicity of clade 2.3.2.1 viruses has been regarded as lower than that of other clades [58]. In our previous studies, a PR8-derived clade 2.3.2.1c recombinant vaccine strain showed less pathogenicity in mice than another PR8-derived recombinant virus containing HA and NA genes from a low pathogenic (LP) A(H5N1) virus [29,59]. Although the amino acid sequences of the two HA proteins are only 89% identical, the HA of the LP A(H5N1) virus has V223 and neither 144NGS nor 158NGS. The higher mouse pathogenicity and replication efficiency of rH5N1-N144S-V223I than other H5N1 recombinant viruses in MDCK and A549 cells may indicate a reduction in mammalian pathogenicity during poultry adaptation to evade immune responses. Poorly glycosylated HA was recognized by an ER stress pathway and induced strong lung injury [60]. The relatively high mammalian pathogenicity of viruses without 144NGS and 158NGS has been verified [23]. As we did not directly compare the pathogenicity of 144NGS- or 158NGS-bearing recombinant viruses, we cannot conclude which virus is more pathogenic in humans. However, a significantly higher frequency of 158NGS- (70.0%) than 144NGS-bearing (1.6%) HA in human cases compared with the frequencies of single 158NGS- (27.2%) or 144NGS-bearing (13.1%) HA in nature may reflect a higher risk of 158NGS than 144NGS in human infection (Table S2).

In conclusion, intensive inoculation of certain types of vaccines may distort natural evolutionary pathways, and the acquired novel adaptive mutations may reduce viral fitness by destabilizing the HA trimer as well as affecting mammalian pathogenicity. Our results may provide clues for future studies to develop more effective vaccine strains and programs that reduce the appearance of antigenic or more human-pathogenic variants.

## Figures and Tables

**Table 1:** Primers used in this study for site-directed mutagenesis.

Primer	Sequence (5′–3′)
N144S-F	GTTCATACCAGGGAAGTTCCTCCTTCTTCAGAAATG
N144S-R	CATTTCTGAAGAAGGAGGAACTTCCCTGGTATGAAC
I223V-F	CACTAGATCCAAAGTAAACGGGCAAAGTGGC
I223V-R	GCCACTTTGCCCGTTTACTTTGGATCTAGTG

**Table 2:** Comparison of 144NGS, 158NGS, and the residue 223 amino acid of hemagglutinin (HA) proteins from early clade 2.3.2, clade 2.3.2.1, clades 2.3.2.1.a, b, and c highly pathogenic (HP) A(H5N1) viruses.

Clade	Strain	Accession No.	144NGS	158NGS	223	Reference
2.3.2	A/duck/China/E319-2/03	AY518362	− ^b^	−	V	[40]
2.3.2.1	A/chicken/Hunan/3/07	GU182142	−	−	V	Direct submission to GenBank
	A/common buzzard/Hong Kong/9213/07	CY036221	+ ^c^	−	V	[6]
2.3.2.1a	A/environment/Chang Sha/25/2009	JN543378	−	−	I	Direct submission to GenBank
2.3.2.1b	A/chicken/Guangxi/S2039/09	KT762439	+	−	V	[41]
2.3.2.1c	A/great crested-grebe/Qinghai/1/2009	CY063318	+	−	I	[42]
	A/ruddy shelduck/Mongolia/X42/09	HM006736	+	−	I	[43]

^a^ H3 numbering; ^b^ −, absence; ^c^ +, presence.

**Table 3:** Mutation profiles (MPs) of 144NGS, 158NGS, and residue 223 in HA genes of A(H5) viruses.

MP	144NGS	158NGS	223	*n* (4189)	Frequency (%)	*n* (711) ^e^	Frequency (%)
1-1	^− a^	−	V	2376	56.7 ^b^		
1-2	−	−	I/others	115 (I(40), others (75))	1.0 (I)		
2-1	−	+ ^a^	V	1122	26.8 ^c^	388	54.6 ^c,f^
2-2	−	+	I/L/R	18/1/1	0.4 (I)	18	2.5
3-1	+	−	V	73	1.7	17	2.4
3-2	+	-	I	478	11.4 ^d^	100	14.1 ^c^
4	+	+	V/I	3/2	0.1		

^a^+/− used for marking presence/absence of N-glycan in HA; ^b^ Significant difference from other MPs (*p* < 0.05); ^c^ Significant difference from MP2-2 (*p* < 0.05); ^d^ Significant difference from MP3-1 (*p* < 0.05); ^e^ Total number of 144NGS/158NGS + 1, 144NGS + 1/158NGS, and 144NGS + 1/158NGS + 1; ^f^ Significant difference from MP3-1 and MP3-2 (*p* < 0.05).

**Table 4:** Gene constellation and viral titers in embryonated chicken eggs (ECEs) of the H5N1 recombinant viruses.

Recombinant Virus	HA	NA	Internal Genes	EID_50_/0.1 mL (log10)
rH5N1	K10-483	K10-483	PR8	7.43 ± 0.28
rH5N1-N144S	K10-483-N144S	K10-483	PR8	6.48 ± 0.23
rH5N1-I223V	K10-483-I223V	K10-483	PR8	7.20 ± 0.17
rH5N1-N144S-I223V	K10-483-N144S-I223V	K10-483	PR8	8.05 ± 0.18 ^a^

^a^ Significant difference from rH5N1-N144S and rH5N1-I223V (*p* < 0.05).

**Table 5:** Replication efficiency of the H5N1 recombinant viruses in mice.

Recombinant Virus	Virus Isolation Rate	EID_50_/0.1 mL (log10) ^a^
rH5N1	3/3	5.75
rH5N1-N144S	3/3	5.25
rH5N1-I223V	3/3	5.00
rH5N1-N144S-I223V	3/3	7.75 ^b^
rPR8	3/3	8.00
Mock (PBS)	0/3	ND ^c^

^a^ EID_50_/0.1 mL was average of three independent replicate experiments; ^b^ Significant difference from other H5N1 recombinant viruses (*p* < 0.05); ^c^ Not detected.

**Table 6:** Comparison of hemagglutination inhibition (HI) titers of egg white against the H5N1 recombinant viruses.

Recombinant Virus	HI Titer of Chicken Egg White	
	Chicken RBC (1%)	Guinea-Pig RBC (1%)
rH5N1	128	64
rH5N1-N144S	64	<8
rH5N1-I223V	128	64
rH5N1-N144S-I223V	128	64

## Supplementary Materials

The following are available online at https://www.mdpi.com/1999-4915/11/10/923/s1, Table S1: Classification and frequency of 144NGS, 158NGS, and their precursors of A(H5) viruses, Table S2: Frequency of 144NGS and 158NGS in HA of A(H5) viruses from laboratory confirmed human cases.

## References

[1] Sonnberg S., Webby R.J., Webster R.G. (2013). Natural history of highly pathogenic avian influenza H5N1. Virus Res..

[2] Lai S., Qin Y., Cowling B.J., Ren X., Wardrop N.A., Gilbert M., Tsang T.K., Wu P., Feng L., Jiang H. (2016). Global epidemiology of avian influenza A H5N1 virus infection in humans, 1997–2015: A systematic review of individual case data. Lancet Infect. Dis..

[3] Smith G., Naipospos T., Nguyen T., de Jong M., Vijaykrishna D., Usman T., Hassan S., Nguyen T., Dao T., Bui N.J.V. (2006). Evolution and adaptation of H5N1 influenza virus in avian and human hosts in Indonesia and Vietnam. Virology.

[4] Le T.H., Nguyen N.T. (2014). Evolutionary dynamics of highly pathogenic avian influenza A/H5N1 HA clades and vaccine implementation in Vietnam. Clin. Exp. Vaccine Res..

[5] Smith G.J., Donis R.O., World Health Organization/World Organisation for Animal Health/Food and Agriculture Organization (WHO/OIE/FAO) H5 Evolution Working Group. Agriculture Organization, H.E.W.G (2015). Nomenclature updates resulting from the evolution of avian influenza A(H5) virus clades 2.1.3.2a, 2.2.1, and 2.3.4 during 2013–2014. Influenza Other Respir. Viruses.

[6] Smith G.J., Vijaykrishna D., Ellis T.M., Dyrting K.C., Leung Y.H., Bahl J., Wong C.W., Kai H., Chow M.K., Duan L. (2009). Characterization of avian influenza viruses A (H5N1) from wild birds, Hong Kong, 2004–2008. Emerg. Infect. Dis..

[7] World Health Organization/World Organisation for Animal Health/Food and Agriculture Organization (WHO/OIE/FAO) H5N1 Evolution Working Group (2014). Revised and updated nomenclature for highly pathogenic avian influenza A (H5N1) viruses. Influenza Other Respir. Viruses.

[8] Laleye A., Joannis T., Shittu I., Meseko C., Zamperin G., Milani A., Zecchin B., Fusaro A., Monne I., Abolnik C. (2018). A two-year monitoring period of the genetic properties of clade 2.3.2.1c H5N1 viruses in Nigeria reveals the emergence and co-circulation of distinct genotypes. Infect. Genet. Evol..

[9] Naguib M.M., Kinne J., Chen H., Chan K.H., Joseph S., Wong P.C., Woo P.C., Wernery R., Beer M., Wernery U. (2015). Outbreaks of highly pathogenic avian influenza H5N1 clade 2.3.2.1c in hunting falcons and kept wild birds in Dubai implicate intercontinental virus spread. J. Gen. Virol..

[10] Nguyen D.T., Jang Y., Nguyen T.D., Jones J., Shepard S.S., Yang H., Gerloff N., Fabrizio T., Nguyen L.V., Inui K. (2017). Shifting Clade Distribution, Reassortment, and Emergence of New Subtypes of Highly Pathogenic Avian Influenza A(H5) Viruses Collected from Vietnamese Poultry from 2012 to 2015. J. Virol..

[11] Bi Y., Chen J., Zhang Z., Li M., Cai T., Sharshov K., Susloparov I., Shestopalov A., Wong G., He Y. (2016). Highly pathogenic avian influenza H5N1 Clade 2.3.2.1c virus in migratory birds, 2014-2015. Virol. Sin..

[12] Wilson I., Skehel J., Wiley D.J.N. (1981). Structure of the haemagglutinin membrane glycoprotein of influenza virus at 3 Å resolution. Nature.

[13] Mair C.M., Ludwig K., Herrmann A., Sieben C. (2014). Receptor binding and pH stability - how influenza A virus hemagglutinin affects host-specific virus infection. Biochim. Biophys. Acta.

[14] Weis W., Brown J., Cusack S., Paulson J., Skehel J., Wiley D.J.N. (1988). Structure of the influenza virus haemagglutinin complexed with its receptor, sialic acid. Nature.

[15] Imai M., Watanabe T., Hatta M., Das S.C., Ozawa M., Shinya K., Zhong G., Hanson A., Katsura H., Watanabe S. (2012). Experimental adaptation of an influenza H5 HA confers respiratory droplet transmission to a reassortant H5 HA/H1N1 virus in ferrets. Nature.

[16] Zhang W., Shi Y., Lu X., Shu Y., Qi J., Gao G.F.J.S. (2013). An airborne transmissible avian influenza H5 hemagglutinin seen at the atomic level. Science.

[17] Swayne D.E., Kapczynski D. (2008). Strategies and challenges for eliciting immunity against avian influenza virus in birds. Immunol. Rev..

[18] Kaverin N.V., Rudneva I.A., Ilyushina N.A., Varich N.L., Lipatov A.S., Smirnov Y.A., Govorkova E.A., Gitelman A.K., Lvov D.K., Webster R.G. (2002). Structure of antigenic sites on the haemagglutinin molecule of H5 avian influenza virus and phenotypic variation of escape mutants. J. Gen. Virol..

[19] Kaverin N.V., Rudneva I.A., Govorkova E.A., Timofeeva T.A., Shilov A.A., Kochergin-Nikitsky K.S., Krylov P.S., Webster R.G. (2007). Epitope mapping of the hemagglutinin molecule of a highly pathogenic H5N1 influenza virus by using monoclonal antibodies. J. Virol..

[20] Wiley D.C., Skehel J.J. (1987). The structure and function of the hemagglutinin membrane glycoprotein of influenza virus. Annu. Rev. Biochem..

[21] Caton A.J., Brownlee G.G., Yewdell J.W., Gerhard W. (1982). The antigenic structure of the influenza virus A/PR/8/34 hemagglutinin (H1 subtype). Cell.

[22] Wiley D.C., Wilson I.A., Skehel J.J. (1981). Structural identification of the antibody-binding sites of Hong Kong influenza haemagglutinin and their involvement in antigenic variation. Nature.

[23] Wang W., Lu B., Zhou H., Suguitan A.L., Cheng X., Subbarao K., Kemble G., Jin H. (2010). Glycosylation at 158N of the hemagglutinin protein and receptor binding specificity synergistically affect the antigenicity and immunogenicity of a live attenuated H5N1 A/Vietnam/1203/2004 vaccine virus in ferrets. J. Virol..

[24] Copeland C.S., Doms R.W., Bolzau E.M., Webster R.G., Helenius A. (1986). Assembly of influenza hemagglutinin trimers and its role in intracellular transport. J. Cell Biol..

[25] Rachakonda P.S., Veit M., Korte T., Ludwig K., Bottcher C., Huang Q., Schmidt M.F., Herrmann A. (2007). The relevance of salt bridges for the stability of the influenza virus hemagglutinin. FASEB J..

[26] DuBois R.M., Zaraket H., Reddivari M., Heath R.J., White S.W., Russell C.J. (2011). Acid stability of the hemagglutinin protein regulates H5N1 influenza virus pathogenicity. PLoS Pathog..

[27] Xiong X., Coombs P.J., Martin S.R., Liu J., Xiao H., McCauley J.W., Locher K., Walker P.A., Collins P.J., Kawaoka Y. (2013). Receptor binding by a ferret-transmissible H5 avian influenza virus. Nature.

[28] Linster M., van Boheemen S., de Graaf M., Schrauwen E.J.A., Lexmond P., Manz B., Bestebroer T.M., Baumann J., van Riel D., Rimmelzwaan G.F. (2014). Identification, characterization, and natural selection of mutations driving airborne transmission of A/H5N1 virus. Cell.

[29] Jang J.W., Lee C.Y., Kim I.H., Choi J.G., Lee Y.J., Yuk S.S., Lee J.H., Song C.S., Kim J.H., Kwon H.J. (2017). Optimized clade 2.3.2.1c H5N1 recombinant-vaccine strains against highly pathogenic avian influenza. J. Vet. Sci..

[30] Lee D.-H., Park J.-K., Youn H.-N., Lee Y.-N., Lim T.-H., Kim M.-S., Lee J.-B., Park S.-Y., Choi I.-S., Song C.-S. (2011). Surveillance and isolation of HPAI H5N1 from wild Mandarin Ducks (Aix galericulata). J. Wildl. Dis..

[31] Hoffmann E., Stech J., Guan Y., Webster R., Perez D.R. (2001). Universal primer set for the full-length amplification of all influenza A viruses. Arch. Virol..

[32] Hoffmann E., Krauss S., Perez D., Webby R., Webster R.G.J.V. (2002). Eight-plasmid system for rapid generation of influenza virus vaccines. Vaccine.

[33] Kim I.H., Kwon H.J., Choi J.G., Kang H.M., Lee Y.J., Kim J.H. (2013). Characterization of mutations associated with the adaptation of a low-pathogenic H5N1 avian influenza virus to chicken embryos. Vet. Microbiol..

[34] Hamilton M., Russo R., Thurston R.J.E.S. (1978). Technology, Trimmed Spearman-Karber method for estimating median lethal concentrations in bioassays. Environ. Sci. Technol..

[35] Lee C.Y., An S.H., Kim I., Choi J.G., Lee Y.J., Kim J.H., Kwon H.J. (2018). Novel mutations in avian PA in combination with an adaptive mutation in PR8 NP exacerbate the virulence of PR8-derived recombinant influenza A viruses in mice. Vet. Microbiol..

[36] Lee C.Y., An S.H., Choi J.G., Lee Y.J., Kim J.H., Kwon H.J. (2018). Acquisition of Innate Inhibitor Resistance and Mammalian Pathogenicity During Egg Adaptation by the H9N2 Avian Influenza Virus. Front. Microbiol..

[37] Matrosovich M.N., Gambaryan A.S. (2012). Solid-phase assays of receptor-binding specificity. Influenza Virus.

[38] Ito T., Suzuki Y., Mitnaul L., Vines A., Kida H., Kawaoka Y.J.V. (1997). Receptor specificity of influenza A viruses correlates with the agglutination of erythrocytes from different animal species. Virology.

[39] An S.H., Kwon H.J. (2019). Freqeuncy of 144N-glycosylation in clade 2.3.2.1c H5N1 viruses. Personal communication.

[40] Lee M., Deng M., Lin Y., Chang C., Shieh H.K., Shiau J., Huang C. (2007). Characterization of an H5N1 avian influenza virus from Taiwan. J. Vet. Microbiol..

[41] Feng X., Wang Z., Shi J., Deng G., Kong H., Tao S., Li C., Liu L., Guan Y., Chen H. (2016). Glycine at position 622 in PB1 contributes to the virulence of H5N1 avian influenza virus in mice. J. Virol..

[42] Li Y., Liu L., Zhang Y., Duan Z., Tian G., Zeng X., Shi J., Zhang L., Chen H. (2011). New avian influenza virus (H5N1) in wild birds, Qinghai, China. Emerg. Infect. Dis..

[43] Kang H.-M., Batchuluun D., Kim M.-C., Choi J.-G., Erdene-Ochir T.-O., Paek M.-R., Sugir T., Sodnomdarjaa R., Kwon J.-H., Lee Y.-J. (2011). Genetic analyses of H5N1 avian influenza virus in Mongolia, 2009 and its relationship with those of eastern Asia. Vet. Microbiol..

[44] Herve P.L., Lorin V., Jouvion G., Da Costa B., Escriou N. (2015). Addition of N-glycosylation sites on the globular head of the H5 hemagglutinin induces the escape of highly pathogenic avian influenza A H5N1 viruses from vaccine-induced immunity. Virology.

[45] WHO/OIE/FAO H5N1 Evolution Working Group (2012). Continued evolution of highly pathogenic avian influenza A (H5N1): Updated nomenclature. Influenza Other Respir. Viruses.

[46] Das S.R., Hensley S.E., David A., Schmidt L., Gibbs J.S., Puigbo P., Ince W.L., Bennink J.R., Yewdell J.W. (2011). Fitness costs limit influenza A virus hemagglutinin glycosylation as an immune evasion strategy. Proc. Natl. Acad. Sci. USA.

[47] Lee C.W., Senne D.A., Suarez D.L. (2004). Effect of vaccine use in the evolution of Mexican lineage H5N2 avian influenza virus. J. Virol..

[48] Ellis T.M., Leung C.Y., Chow M.K., Bissett L.A., Wong W., Guan Y., Malik Peiris J.S. (2004). Vaccination of chickens against H5N1 avian influenza in the face of an outbreak interrupts virus transmission. Avian Pathol..

[49] Chen H., Bu Z. (2009). Development and application of avian influenza vaccines in China. Curr. Top. Microbiol. Immunol..

[50] Li C., Bu Z., Chen H. (2014). Avian influenza vaccines against H5N1 ‘bird flu’. Trends Biotechnol..

[51] FAO-OIE-WHO (2011). FAO-OIE-WHO Technical Update: Current evolution of avian influenza H5N1 viruses. Avian Flu Diary.

[52] Nguyen L.T., Firestone S.M., Stevenson M.A., Young N.D., Sims L.D., Chu D.H., Nguyen T.N., van Nguyen L., Thanh Le T., van Nguyen H. (2019). A systematic study towards evolutionary and epidemiological dynamics of currently predominant H5 highly pathogenic avian influenza viruses in Vietnam. Sci. Rep..

[53] FAO (2011). FAO-AIDE News, Update on the avian influenza situation. Flu China.

[54] Kim J.I., Lee I., Park S., Hwang M.W., Bae J.Y., Lee S., Heo J., Park M.S., Garcia-Sastre A., Park M.S. (2013). Genetic requirement for hemagglutinin glycosylation and its implications for influenza A H1N1 virus evolution. J. Virol..

[55] Lanni F., Beard J. (1948). Inhibition by egg-white of hemagglutination by swine influenza virus. Proc. Soc. Exp. Biol. Med..

[56] An S.H., Lee C.Y., Hong S.M., Choi J.G., Lee Y.J., Jeong J.H., Kim J.B., Song C.S., Kim J.H., Kwon H.J. (2019). Bioengineering a highly productive vaccine strain in embryonated chicken eggs and mammals from a non-pathogenic clade 2.3.4.4 H5N8 strain. Vaccine.

[57] De Vries R.P., Zhu X., McBride R., Rigter A., Hanson A., Zhong G., Hatta M., Xu R., Yu W., Kawaoka Y. (2014). Hemagglutinin receptor specificity and structural analyses of respiratory droplet-transmissible H5N1 viruses. J. Virol..

[58] Creanga A., Hang N.L.K., Cuong V.D., Nguyen H.T., Phuong H.V.M., Thanh L.T., Thach N.C., Hien P.T., Tung N., Jang Y. (2017). Highly Pathogenic Avian Influenza A(H5N1) Viruses at the Animal-Human Interface in Vietnam, 2003–2010. J. Infect. Dis..

[59] Kim I.H., Kwon H.J., Park J.K., Song C.S., Kim J.H. (2015). Optimal attenuation of a PR8-derived mouse pathogenic H5N1 recombinant virus for testing antigenicity and protective efficacy in mice. Vaccine.

[60] Hrincius E.R., Liedmann S., Finkelstein D., Vogel P., Gansebom S., Samarasinghe A.E., You D., Cormier S.A., McCullers J.A. (2015). Acute Lung Injury Results from Innate Sensing of Viruses by an ER Stress Pathway. Cell Rep..

