# Supplementary material for: Novel Mutations Evading Avian Immunity around the Receptor Binding Site of the Clade 2.3.2.1c Hemagglutinin Gene Reduce Viral Thermostability and Mammalian Pathogenicity
Source: Viruses. 2019 Oct 9;11(10):923. doi: 10.3390/v11100923 (PMC6832455; doi:10.3390/v11100923)
Supplement: Supplementary file 1 [file viruses-11-00923-s001.pdf]

Table S1. Classification and frequency of 144NGS, 158NGS, and their precursors of A(H5) viruses.

No. of mutations to become 144NGS (4075)				No. of mutations to become 158NGS (4065)				
0 (551)	+1(658)	+2 (2489)	+3 (377)	0 (1141)	+1 (1824)	+2 (996)	+3 (104)	+4 (2)
NSS (551)	KSS (550)	RSS (1812)	GPS (130)	NNT (490)	NNA(927)	DNA(841)	DNV(56)	ENV(1)
	NPS (36)	TPS (292)	MPS (92)	NDT (49)	NDA(596)	NNV(54)	GNA(29)	GNE(1)
	SSS (55)	SPS (115)	RPS (68)	NST (563)	NSA(217)	DDA(21)	DGV(6)	
	TSS (10)	ASS (74)	VPS (39)	NSS (30)	DNT(53)	DSA(18)	DDV(4)	
	SHS (1)	KPS (73)	APS (20)	NNS (5)	NAA(9)	SNA(18)	DNM(3)	
		QSS (51)	EPS (19)	NGT (2)	NGA(8)	NNE(16)	QNA(2)	
		GSS (15)	RSA (6)	NDS (1)	DST(3)	NDV(10)	INV(1)	
		MSS (14)	ASA (1)	NHT (1)	NNI(2)	SDA(6)	DNE(1)	
		GLS (12)	KPF (1)	NTT (1)	NSI(2)	DSS(3)	ANA(1)	
		ESS (8)	RSF (1)		NDK(1)	GST(2)	GDA(1)	
		ISS (7)			NDP(1)	NDE(1)		
		RTS (6)			NNK(1)	NNQ(1)		
		RAS (3)			NNM(1)	DTA(1)		
		VSS (2)			DNS(1)	ENA(1)		
		DPS (1)			SNS(1)	SNT(1)		
		ELS (1)			SST(1)	YNA(1)		
		GRS (1)						
		MLS (1)				KNN(1)		
		RFS (1)						
% of 144NGS	% of precursor 144NGS			% of 158NGS	% of precursor 158NGS			
13.5% (551/4075)	86.5% (3524/4075)			28.1% (1141/4065)	71.9% (2924/4065)			

Table S2. Frequency of 144NGS and 158NGS in HA of A(H5) viruses from laboratory-confirmed human cases.

NGS	Human (n=513)	Avian (n=4,189)
144NGS	1.6%	13.1%[a]
158NGS	70.0%[b]	27.2%
144NGS/158NGS	0.4%	0.1%
None	28.1%	57.7%[c]

[a] significant difference with 144NGS in human ($p<0.05$).

[b] significant difference with 158NGS in avian ($p<0.05$).

[c] significant difference with none in human ($p<0.05$).
